# Biodistribution and intestinal inflammatory response following voluntary oral intake of silver nanoparticles by C57BL/6J mice

**DOI:** 10.1007/s00204-023-03558-5

**Published:** 2023-08-18

**Authors:** Adelaide Sousa, Rui Azevedo, Vera Marisa Costa, Sara Oliveira, Inês Preguiça, Sofia Viana, Flávio Reis, Agostinho Almeida, Paulo Matafome, Patrícia Dias-Pereira, Félix Carvalho, Eduarda Fernandes, Marisa Freitas

**Affiliations:** 1 LAQV, REQUIMTE, Laboratory of Applied Chemistry, Department of Chemical Sciences, Faculty of Pharmacy, University of Porto, Rua de Jorge Viterbo Ferreira N.º 228, 4050-313 Porto, Portugal; 2 UCIBIO, REQUIMTE, Laboratory of Toxicology, Department of Biological Sciences, Faculty of Pharmacy, University of Porto, 4050‑313 Porto, Portugal; 3 Associated Laboratory i4HB – Institute for Health and Bioeconomy, Faculty of Pharmacy, University of Porto, Porto, Portugal; 4 Coimbra Institute of Clinical and Biomedical Research (iCBR), Faculty of Medicine and Center for Innovative Biomedicine and Biotechnology (CIBB), University of Coimbra, Coimbra, Portugal; 5 Clinical Academic Center of Coimbra, Coimbra, Portugal; 6 Instituto Politécnico de Coimbra, Coimbra Health School (ESTeSC), Coimbra, Portugal; 7 ICBAS School of Medicine and Biomedical Sciences, University of Porto (ICBAS-UP), Porto, Portugal

**Keywords:** Oral intake, Biodistribution, Subacute toxicity, Intestinal inflammation, NF-кB inflammatory pathway, Cytokines and chemokines

## Abstract

Silver nanoparticles (AgNP) are among the most widely commercialized nanomaterials globally, with applications in medicine and the food industry. Consequently, the increased use of AgNP in the food industry has led to an unavoidable rise  in human exposure to these nanoparticles. Their widespread use raises concerns about potential hazards to human health, specifically their intestinal pro-inflammatory effects. Thus, the main objective of this study was to evaluate the biological effects of two subacute doses of 5 nm polyvinylpyrrolidone (PVP)-AgNP in C57BL/6J mice. One mg/kg body weight or 10 mg/kg bw was provided once a day for 14 days, using a new technology (HaPILLness) that allows voluntary, stress-free, and accurate oral dosing. It was observed that after oral ingestion, while AgNP is biodistributed throughout the entire organism, most of the ingested dose is excreted in the feces. The passage and accumulation of AgNP throughout the intestine instigated a prominent inflammatory response, marked by significant histological, vascular, and cellular transformations. This response was driven by the activation of the nuclear factor-кB (NF-кB) inflammatory pathway, ultimately leading to the generation of multiple cytokines and chemokines.

## Introduction

In recent decades, nanoparticles, especially silver nanoparticles (AgNP), have gained wide importance with ubiquitous presence in our daily life. They are currently applied in a multitude of sectors, from medicine to the food industry, due to their unique physicochemical and biological properties (Gupta and Xie 2018; Sousa et al. 2022). Despite their wide range of applications, AgNP acquired special relevance in the food sector. In fact, due to their antibacterial properties, the direct or indirect incorporation of AgNP in food by the food industry, in any stage of the food development process from food cultivation to industrial product manufacture, including processing and packaging, improves food quality, safety, and nutritional attributes (Paidari et al. 2021; Sousa et al. 2022).

Despite the numerous benefits of AgNP applications, they can also raise some concerns, as they can migrate from containers to food, being inadvertently ingested. This frequent contact with AgNP, together with their ultra-small sizes, may result in an increase of their bioavailability and systemic exposure (Zorraquín-Peña et al. 2020; Paidari et al. 2021). In addition to surface charge, shape, concentration, and exposure duration, the size and coating of AgNP are important factors that can affect their toxicity and inflammatory effects (Tsatsakis 2021). There are several studies showing that smaller AgNP have higher toxicity when compared to larger ones. This can be attributed to the fact that smaller AgNP have a larger surface area to volume ratio. As a result, they can interact more effectively with biological molecules, leading to enhanced cellular uptake, which may result in increased toxicity (Guo et al. 2016; Soares et al. 2016; Cho et al. 2018; Ferdous and Nemmar 2020; Sousa et al. 2022, 2023). Coatings, as polyvinylpyrrolidone (PVP), are often used to stabilize AgNP and prevent aggregation. However, some AgNP coatings can also affect their toxicity. (Guo et al. 2016; Soares et al. 2016; Cho et al. 2018; Ferdous and Nemmar 2020; Sousa et al. 2022, 2023). The human dietary intake of silver (Ag), resulting from the widespread use of AgNP, has been estimated to be 70–90 μg/day (Zorraquín-Peña et al. 2020). However, considering the increased use of nanoparticles from this metal in the food industry, a substantial increase in the level of exposure is expected (Mathur et al. 2018; Zorraquín-Peña et al. 2020). Therefore, the risks associated with their potential migration into food and, consequently, their unpredicted harmful effects, pose a safety concern (Tsatsakis 2021; Zorraquín-Peña et al. 2020; Paidari et al. 2021; Sousa et al. 2022). Since the gastrointestinal tissues (GIT) are the first to be exposed to dietary AgNP, it can be assumed that they are particularly susceptible to their toxicity and possible pro-inflammatory effects (McClements and Xiao 2017; Zorraquín-Peña et al. 2020; Paidari et al. 2021). The perpetuation of an inflammatory response in the GIT can lead to several modifications in the epithelial barrier, activation of immune intestinal cells and their infiltration in the intestinal mucosa, upregulation of transcription factors such as nuclear factor kappa B (NF-кB), and the release of inflammatory mediators, including cytokines and chemokines (Friedrich et al. 2019, Lamas et al. 2020, Peng et al. 2020, Ghebretatios et al. 2021, Sousa et al. 2022). Ultimately, these events may contribute to the development or worsening of various gut-related diseases, including inflammatory bowel diseases (IBD) (Crohn's disease and ulcerative colitis) (Guan 2019; Lamas et al. 2020, Ghebretatios et al. 2021).

Thus, this study aimed to evaluate the biodistribution of subacute administration of 5 nm PVP-AgNP in C57BL/6 J mice. The AgNP were given through the oral route, at doses of 1 mg/kg bw (body weight) or 10 mg/kg bw, once a day for 14 days, using a new technology (HaPILLness) that allows voluntary, stress-free, and accurate oral dosing. Thus, the in vivo biodistribution of AgNP was assessed, as well as the AgNP-induced harmful effects in the intestine, through histopathological analysis and the determination of plasma and tissue levels of a set of inflammatory cytokines. The tissue expression levels of pro-inflammatory proteins involved in the NF-кB signaling pathway, namely IкBα and phospho-IкBα were also analyzed.

## Materials and methods

### Chemicals

All solvents and chemicals were of analytical grade. Pure PVP-coated AgNP (5 nm) were obtained from nanoComposix (San Diego, CA). Ultrapure deionized water was obtained by an Arium® pro system (Sartorius, Goettingen, DE), TraceSELECT™ nitric acid (≥ 65% v/v) (Honeywell Fluka, Fisher Scientific, Waltham, MA, USA), TraceSELECT™ hydrochloric acid (30–32% v/v) (Honeywell Fluka) and Primar™ hydrogen peroxide (≥ 30–32% v/v) (Fisher Chemical, Loughborough, UK) were used throughout. Proteases inhibitor cocktail (Mini Complete™, Roche), phosphatases inhibitor cocktail (PhosSTOP, Roche), phosphate-buffered saline (PBS), hematoxylin (Mayer’s solution), and eosin were obtained from Merck (Darmstadt, DE). The commercial BD™ Cytometric Bead Array (CBA) Mouse Inflammatory Cytokines Kit was obtained from BD Biosciences (San Diego, CA, USA). Primary antibodies targeting IкBα and phospho-IкBα were obtained from Cell Signaling (Danvers, MA, USA) and anti-mouse IgG, HRP-conjugated, secondary antibodies were obtained from Santa Cruz Biotechnologies (Santa Cruz, CA, USA). DC™ Protein Assay kit II and Clarity ECL Western Blot Substrate kit were obtained from BioRad Laboratories (Hercules, CA, USA).

### Animals and experimental design

The study was performed according to good practices of animal handling, with the approval of the Institutional Animal Care and Use Committee (ORBEA process number 13/2018) and the procedures performed by licensed users by the Federation of Laboratory Animal Science Associations (FELASA), as stated in the guidelines from the Portuguese Law on Experimentation with Laboratory Animals (Decree-Law nº 113/2013) and the Directive 2010/63/EU of the European Parliament for the Protection of Animals Used for Science Purpose.

Male 12-week-old C57BL/6J mice weighing 21–27 g were purchased from Charles River Laboratories (France). Animals were kept under standard conditions—2 animals *per* cage, with temperature at 22–24 °C, and 50–60% humidity, and standard light cycle (12-h light/12-h darkness), with water and standard food (4RF21, Mucedola, Italy) ad libitum. All animals were allowed to acclimatize to the animal room conditions and trained for 2 weeks by the handler for voluntary feeding. Then, the animals were randomly divided into experimental groups. Animal welfare was assessed during the entire period by experienced veterinarians.

### Semi-solid formulation for voluntary oral intake of silver nanoparticles

The voluntary oral intake of 5 nm PVP-AgNP was carried out using a new oral dosing formulation technology (HaPILLness) that allows the production of semi-solid matrices for the incorporation of testing compounds for oral administration. It allows voluntary, stress-free, and precise oral dosing due to rodents’ voluntary acceptance and full consumption. Formula composition includes a diluent, thickening, and flavor masking agents along with digestible sweeteners (under patent application PCT/IB2021/053124).

**Fig. 1:**
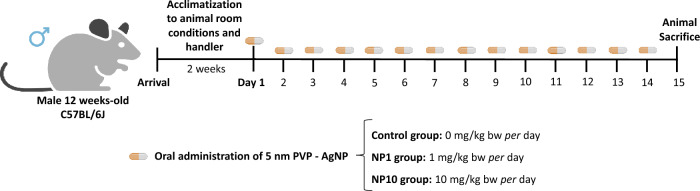
Schematic representation of the experimental design. **Control:** Untreated animals; **NP1:** Animals treated with 5 nm PVP-AgNP at 1 mg/kg bw; **NP10:** Animals treated with 5 nm PVP-AgNP at 10 mg/kg bw. For oral dosing, two animals simultaneous consume the semi-solid matrices in their home-cage where a partition is inserted only for the duration of consumption. Optimal voluntary acceptance occur < 5 min. At the end, all animals return to their home-cage

Mice were randomly divided into three experimental groups (*n* = 5–6/group): control (*n* = 5) and animals dosed with pills with 5 nm PVP-AgNP at 1 mg/kg bw (*n* = 5) or 10 mg/kg bw (*n* = 6) for 14 days. The control group was exposed to the same procedure for the administration of empty semi-solid matrices (without 5 nm PVP-AgNP) for 14 days (Fig. 1).

### Animal behavior, body, and organ weights

The animals were supervised during the 14 days of treatment and all mice appeared healthy and no abnormal behavior and/or clinically relevant illness were observed. Body weight was weekly monitored. The ratio of organ/body weight was calculated. No significant differences in body and organ weights were observed between treated and control mice.

### Blood and organ collection

Animals were anesthetized with an i.p. injection of ketamine/chlorpromazine at the end of treatment with AgNP. Blood was immediately collected by cardiac puncture into Vacutainer^TM^ EDTA tubes (BD Biosciences, Franklin Lakes, NJ, USA) and centrifuged at 2200 g, for 15 min, at 4 °C, and plasma was separated and stored at − 80 °C for posterior cytokine analysis. After euthanasia by cervical displacement, the brain, lungs, liver, kidneys, heart, spleen, adipose tissue, testes, duodenum, ileum, and colon were collected, weighed, washed in ice-cold PBS solution, and placed into decontaminated (acid-washed) polypropylene tubes. The feces were also collected from the intestine into decontaminated (acid-washed) polypropylene tubes.

For histological analysis, an ~ 1 cm section of duodenum, ileum, or colon was taken, washed in Krebs buffer to remove all remaining feces from the gut and fixed in 10% formaldehyde solution. For the biodistribution assessment, inflammatory cytokine production and western blotting analysis, the remaining organs/tissues and feces were immediately frozen at − 80 °C, until use.

### Determination of total silver content in organs and feces

#### Microwave-assisted acid digestion

An ETHOS™ EASY microwave digestion platform (Milestone, Sorisole, Italy) equipped with an SK-15 easyTEMP high-pressure rotor was used for closed vessel acid digestion of the samples. Organs or tissues (brain, lungs, liver, kidneys, heart, spleen, adipose tissue, testes, duodenum, ileum, colon) and feces were previously dried to a constant weight (100 °C, 24 h). Samples were then placed in the PTFE vessels of the microwave oven and 9 mL of HNO_3_, 2 mL of H_2_O_2_ and 1 mL of HCl were added. Then, the vessels were closed, placed in the microwave oven and the following program was applied: gradual temperature increase to 210 °C for 20 min, followed by 15 min at 210 °C and 20 min of cooling. Sample blanks were prepared under the same conditions. Results were calculated as μg of Ag *per* kg of tissue analyzed. At least 3 digestions of the same tissue type were conducted.

#### Elemental analysis

Determination of Ag in the solutions was done by inductively coupled plasma-mass spectrometry (ICP-MS) using an iCAP™ Q instrument (Thermo Fisher Scientific, Bremen, DE) equipped with a Meinhard® (Golden, CO, USA) TQ + quartz concentric nebulizer, a Peltier cooled, high-purity quartz, baffled cyclonic spray chamber, and a demountable quartz torch with a 2.5 mm id quartz injector. The interface was two Ni cones (sampler and skimmer). High-purity argon (99.9997%) supplied by Gasin (Leça da Palmeira, Portugal) was used as nebulizer, auxiliary, and plasma gas. Before each analytical run, the instrument was tuned for maximum sensitivity and signal stability and minimum formation of oxides and double-charged ions. The main operating parameters of the ICP-MS instrument were: nebulizer gas flow, 1.11 L/min; auxiliary gas flow, 0.79 L/min; plasma gas flow, 14.0 L/min; radiofrequency generator power, 1550 W; dwell time, 30 ms. A 9-point calibration curve was created (0.05, 0.10, 0.20, 0.50, 1.0, 5.0, 10.0, 20.0, and 50.0 µg/L of Ag) with calibration standards prepared by appropriate dilution of a multi-element standard solution (Periodic Table Mix 1, 10 mg/L in 10% HNO_3_ w/w, Sigma-Aldrich, Buchs, Switzerland). Both samples and calibration standards were diluted 1:5 with a diluent consisting of 2% (v/v) HNO_3_, 2% (v/v) HCl and 10 μg/L Rh internal standard (from Rh ICP Standard, 1000 mg/L in 3% HNO_3_, Honeywell Fluka). After thorough mixing on a vortex mixer, the diluted samples and calibration standards were presented to the ICP-MS instrument using a CETAC ASX-520 autosampler (Teledyne CETAC Technologies, Omaha, NE, USA). The elemental isotopes (*m/z* ratios) ^109^Ag and ^103^Rh (IS) were monitored for analytical purposes.

#### Analytical quality control

For analytical quality control purposes, the certified reference material (CRM) DOLT-4 (Fish Liver Certified Reference Material for Trace Metals), provided by the National Research Council of Canada (NRCC, Ottawa, CA), was digested and analyzed under the same conditions as for the samples.

### Histological analysis

To evaluate the effect of AgNP on the GIT, basic histological analysis of the duodenum, ileum, and colon of treated and untreated animals was performed. For that purpose, after fixation, tissue samples were processed routinely and embedded in paraffin wax. Serial sections with 2 µm thickness were cut and stained with hematoxylin–eosin. The slides were examined with an optical microscope (Nikon, model Eclipse E600, Nikon Instruments, Miami, FL, USA), and photographed in two or three representative regions with objective lenses of 4×, 10× and 20× (magnification of 40×, 100×, and 200×). The obtained images were used to measure the thickness of the mucosa, submucosa and the whole intestinal wall. In addition, villi width and crypt depth were assessed in samples of duodenum and ileum. All the measures were carried out always by the same person, using the free ImageJ® software 1.53t. For each sample, the layer thickness was measured in five different locations and averaged.

### Evaluation of inflammatory cytokines

The production of interleukin (IL)-6, IL-10, monocyte chemoattractant protein-1 (MCP-1), interferon-γ (IFN-γ), tumor necrosis factor (TNF), and IL-12p70 was measured in plasma and tissues, namely colon, ileum, and duodenum homogenates from untreated and treated animals. For this purpose, a flow cytometry detection kit [BD™ Cytometric Bead Array (CBA) Mouse Inflammatory Cytokines Kit, BD Biosciences] was used, following the manufacturer’s instructions. Briefly, aliquots of plasma or homogenates from the colon, ileum, or duodenum were incubated with the mixed capture beads for 2 h at room temperature, protected from light. After this incubation period, wash buffer was added to the test tubes and centrifuged at 200 g for 5 min. The supernatant was then aspirated and discarded, and wash buffer was again added to the test tubes to resuspend the pellet. The samples were immediately analyzed in a BD Accuri ™ C6 flow cytometer (BD Biosciences), using the red channel (FL3). Results were expressed as the mean fluorescence intensity (MFI).

### Western Blot analysis

Proteins were isolated after homogenizing the entire samples of the colon, ileum or duodenum in lysis buffer [50 mM Tris–HCl, pH = 7.5; 150 mM NaCl; 5 mM EDTA; 1% Triton X-100; 0.5% sodium deoxycholate; 0.1% sodium dodecyl sulfate (SDS); ultrapure H_2_O] containing a cocktail of protease inhibitors and a cocktail of phosphatases inhibitors. The extracts were then centrifuged (18,000 g, 10 min, 4 °C). The supernatant fractions were collected for blotting analysis. Protein levels were quantified using the DC™ Protein Assay kit II, according to the manufacturer’s instructions, using bovine serum albumin as a standard.

In the western blotting analysis, proteins (50 μg/ well) were separated by SDS-PAGE and transferred to polyvinylidene fluoride (PVDF) membranes. The membranes were blocked with 5.0% skim milk powder for 1 h at room temperature and subsequently labeled with the following antibodies: mouse monoclonal anti-mouse IκBα and mouse monoclonal anti-mouse phospho-IκBα. Mouse anti-β-actin monoclonal antibody was used to detect β-actin as loading control. Anti-mouse and anti-rabbit HRP-conjugated secondary antibodies were used as secondary antibodies. Immune complexes were detected with the Clarity ™ ECL Western Blot substrate using a ChemiDoc Imaging System (Biorad). The obtained bands were analyzed using the CLICS software (version 7.0, Total Lab, Gosforth, UK). The results were normalized by calculating the ratio between the intensities of the bands corresponding to the protein of interest and β-actin that was used as the loading control.

### Statistical analysis

Results were expressed as the mean ± standard error of the mean (SEM). Statistical comparisons were made between the control and the two concentrations of 5 nm PVP-coated AgNP-treated groups, using the one-way analysis of variance (ANOVA), followed by Fishers’s LSD test. Statistical significance was accepted at *p* < 0.05. When *p* > 0.05 but < 0.1, a tendency is considered. The GraphPad Prism™ version 7.00 (GraphPad, San Diego, CA) software was used to create graphs and perform statistical tests.

## Results

### Determination of total silver content in organs and feces

To assess the biodistribution of 5 nm PVP-AgNP, total Ag levels in the brain, lungs, liver, kidneys, heart, spleen, adipose tissue, testes, duodenum, ileum, colon, and feces of treated mice (5 nm PVP-AgNP at 1 and 10 mg/kg bw) and controls were measured.

The results obtained for the biodistribution of 5 nm PVP-AgNP in C57BL/6J mice are summarized in Table 1.

**Table 1:** Silver biodistribution in C57BL/6J mice organs/tissues, 14 days after oral administration of 5 nm PVP-AgNP (1 and 10 mg/kg bw)

Organ/tissue	Ag content (μg/kg)		
	Control group	NP1	NP10
Brain	18 ± 14	104 ± 19 ^**^	162 ± 14 ^*** #^
Lungs	13 ± 3	402 ± 44 ^***^	681 ± 46 ^**** ##^
Liver	1 ± 0	32 ± 10 ^p=0,06^	56 ± 13 ^**^
Kidneys	3 ± 1	5 ± 1	14 ± 3 ^*** ##^
Heart	7 ± 1	21 ± 7 ^****^	20 ± 1 ^*** ##^
Spleen	10 ± 4	36 ± 14	98 ± 29 ^*^
Adipose tissue	2 ± 0	3 ± 1	3 ± 1
Testes	6 ± 2	74 ± 57	98 ± 30
Duodenum	11 ± 2	15 ± 5	38 ± 17 ^p=0.08^
Ileum	10 ± 2	26 ± 13	49 ± 13 ^*^
Colon	17 ± 6	17 ± 3	45 ± 7 ^** ##^
Feces	45 ± 11	223 ± 38	770 ± 149 ^**** ###^

Values are presented as mean $\pm$ SEM, n ≥ 3 *per* group. **Control:** Untreated animals; **NP1:** Animals treated with 5 nm PVP-AgNP at 1 mg/kg bw; **NP10:** Animals treated with 5 nm PVP-AgNP at 10 mg/kg bw. The mass concentration of Ag in the formulation administered is 5.19 mg/mL. ****p < 0.0001, ***p < 0.001, **p < 0.01 and *p < 0.05 when compared to control (untreated animals). ^###^p < 0.001, ^##^p < 0.01 and ^#^p < 0.05 when compared to mice administered with 1 mg/kg bw of 5 nm PVP-AgNP

It may be noted that Ag was present only in ultra-trace amounts in the untreated animals (control group). In contrast, treated animals accumulated Ag in all organs/tissues, generally in a concentration-dependent manner, showing that 5 nm PVP-AgNP were distributed throughout the organism. With the highest dose (10 mg/kg bw), this accumulation of Ag was not significant in adipose tissue, testes, and duodenum (Table 1). In all other organs/tissues analyzed (brain, lungs, liver, kidneys, heart, spleen, ileum, colon, and feces), Ag accumulation reached statistically higher levels when compared with controls. At the lowest dose (1 mg/kg bw), the difference was statistically significant only for the brain, lungs, and heart. For both tested doses (1 and 10 mg/kg bw), the organs that showed the highest accumulation of Ag were the lungs (Table 1).

### Histological analysis

As this study aimed to address the intestinal effect of these AgNP, histological examination was performed in different intestinal segments.

**Fig. 2:**
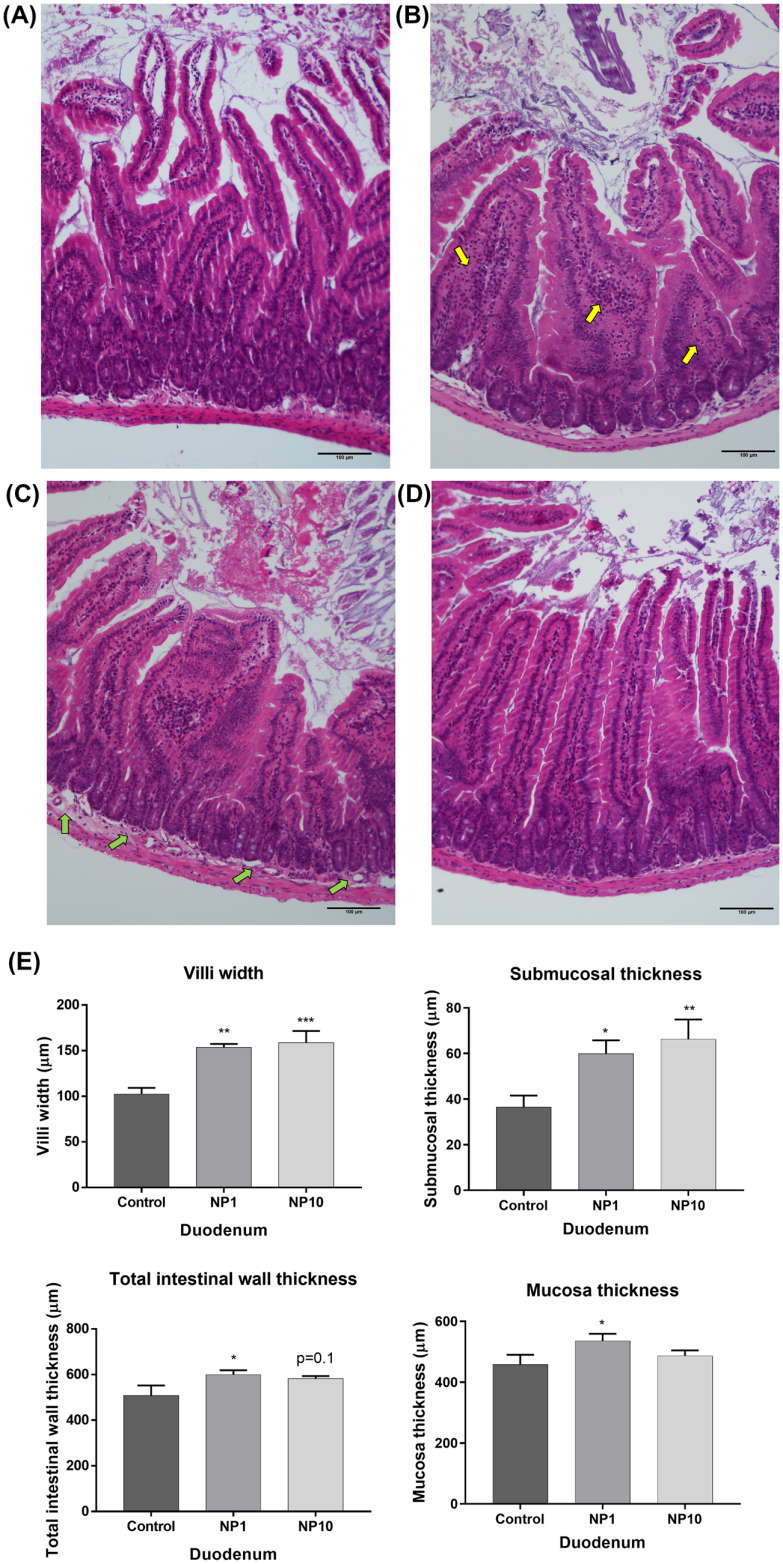
Microphotography of the duodenum of controls **(A)** and animals treated with 5 nm PVP-AgNP at 10 mg/kg bw **(B–D)**. Note the enlargement of the intestinal villi due to the mononuclear inflammatory infiltrate (yellow arrows) in the lamina propria in figure **(B)**, as well as the swelling of the submucosa (green arrows) in figure **(C)**. It is also noticeable a slight enlargement of the whole intestinal wall and mucous membrane in **(D)**. Histomorphometric evaluation of duodenum **(E)**: villi width, submucosal thickness, total intestinal wall thickness, mucosa thickness. **Control:** Untreated animals; **NP1:** Animals treated with 5 nm PVP-AgNP at 1 mg/kg bw; **NP10:** Animals treated with 5 nm PVP-AgNP at 10 mg/kg bw. Values are presented as mean ± SEM, n ≥ 5 per group. ***p < 0.001, **p < 0.01 and *p < 0.05 when compared to control (untreated animals)

**Fig. 3:**
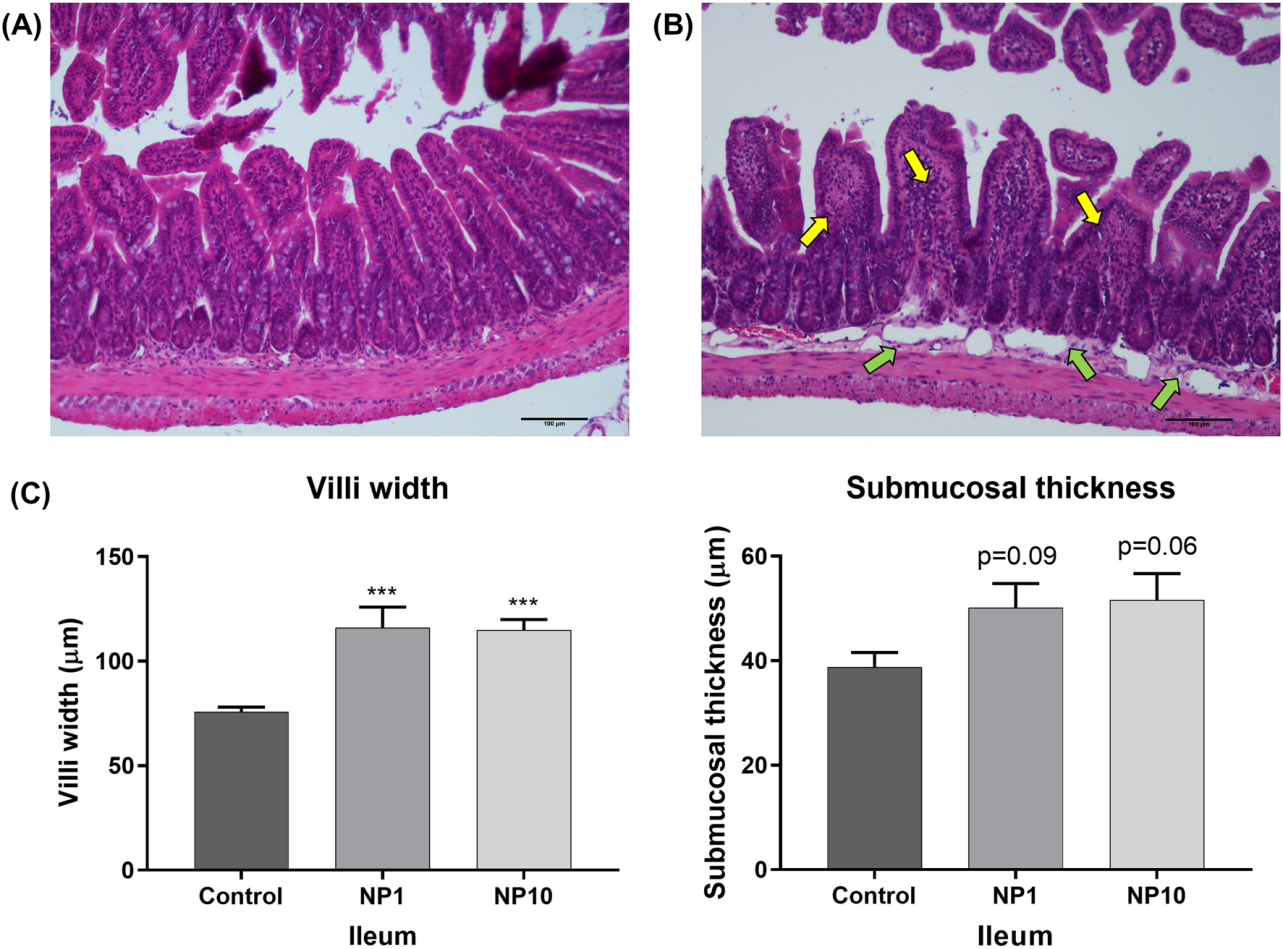
Microphotography of the ileum of controls **(A)** and animals treated with PVP-AgNP at 10 mg/kg bw **(B)**. Note the villi enlargement (yellow arrows) and the submucosa thickening due to vascular ectasia (green arrows) and edema in **(B)**. Histomorphometric evaluation of ileum **(C)**: villi width and submucosal thickness. **Control:** Untreated animals; **NP1:** Animals treated with 5 nm PVP-AgNP at 1 mg/kg bw; **NP10:** Animals treated with 5 nm PVP-AgNP at 10 mg/kg bw. Values are presented as mean ± SEM, n ≥ 5 per group. ***p < 0.001 when compared to control (untreated animals)

The analysis revealed a discrete diffuse mononuclear inflammatory infiltrate, composed mainly of lymphocytes and macrophages, in the mucosa of the duodenum (Figs. 2A vs 2B) and ileum (Figs. 3A vs 3B), resulting in villi enlargement, in most animals from both PVP-AgNP-treated groups (3/5 and 5/6 of 1 and 10 mg/kg bw-treated groups, respectively). The duodenal histomorphometry (Fig. 2E) showed a significative and concentration-dependent increase in villi width in animals treated with 1 mg/kg bw (154 ± 4 μm) and with 10 mg/kg bw (159 ± 13 μm), in comparison with untreated animals (102 ± 7 μm). In the ileum (Fig. 3C), villi width ranged from 76 ± 2 μm (control) to 116 ± 10 μm (1 mg/kg bw-treated animals) and 115 ± 5 μm (10 mg/kg bw-treated animals). Furthermore, most animals treated with PVP-AgNP (4/5 and 5/6 of 1 and 10 mg/kg bw-treated groups, respectively) exhibited edema and vascular ectasia in duodenal (Fig. 2A vs C) and ileal (Fig. 3A vs B) submucosa. The duodenum of animals treated with 1 mg/kg bw and 10 mg/kg bw presented a higher submucosal thickness, about 1.6 (60 ± 6 μm) and 1.8 times (66 ± 9 μm), respectively, than the untreated group (37 ± 5 μm) (Fig. 2E). On the other hand, the increase in the ileum submucosal thickness was not as significant, with only a tendency for a slight increase in animals treated with both concentrations of PVP-AgNP (50 ± 5 μm and 52 ± 5 μm for animals treated with 1 and 10 mg/kg bw, respectively, in comparison to 39 ± 3 μm for untreated animals) (Fig. 3C). Histomorphometry also showed a slight increase in the total thickness of the duodenal intestinal wall and of the mucosa (Fig. 2A vs D). Interestingly, this increase was only found in 1 mg/kg bw-treated animals, which presented an intestinal wall and mucosa thickness of 600 ± 20 μm and 536 ± 23 μm, respectively. On the other hand, no significant differences were found in crypt depth for duodenum or ileum in PVP-AgNP-treated animals compared to control (untreated) animals (data not shown).

**Fig. 4:**
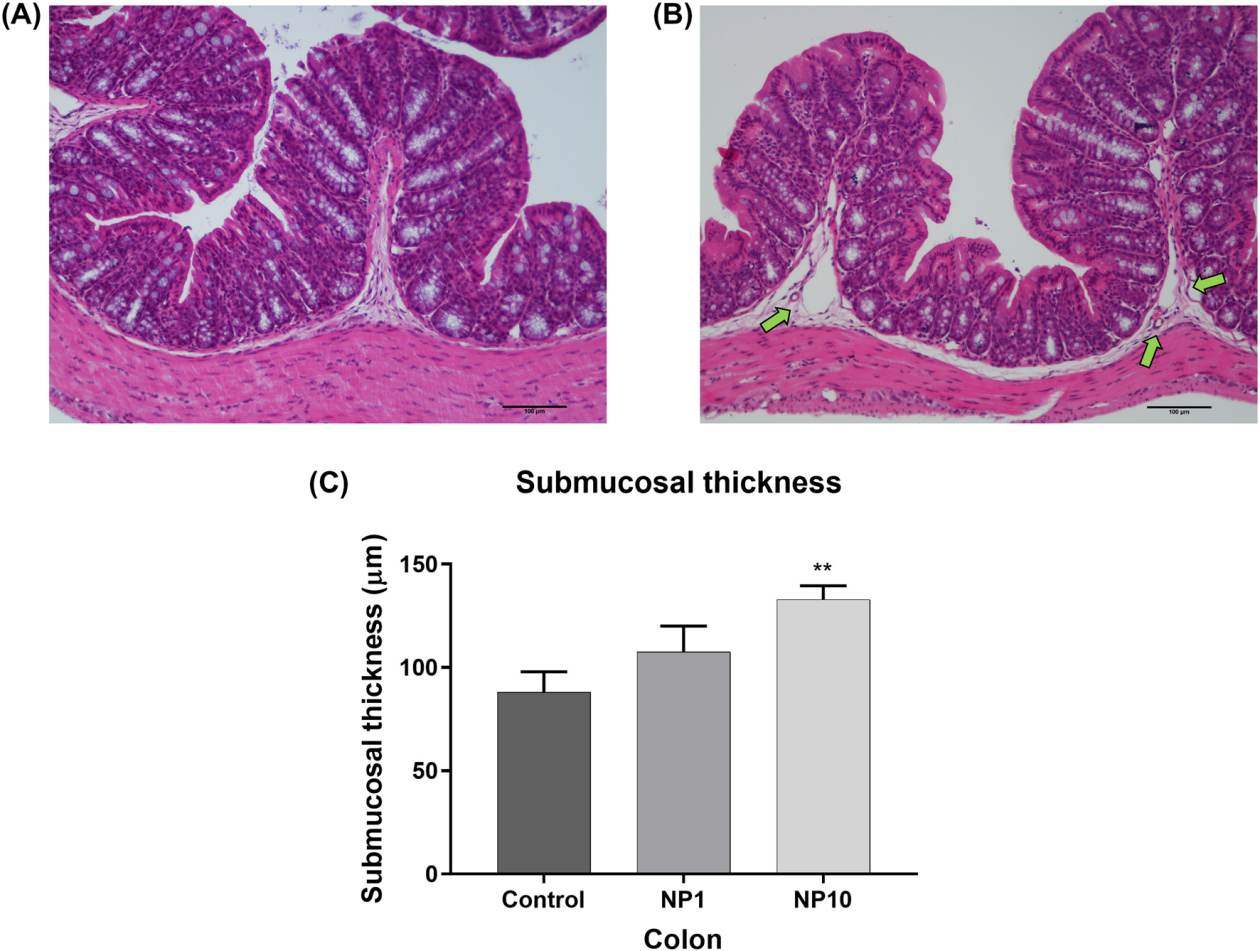
Microphotography from **the colon** of control **(A)** and PVP-AgNP (10 mg/kg bw)-treated animals **(B)**. Note the submucosa thickening due to vascular ectasia (green arrows) and edema in **(B)**. Histomorphometric evaluation of colon **(C)**: submucosal thickness. **Control:** Untreated animals; **NP1:** Animals treated with 5 nm PVP-AgNP at 1 mg/kg bw; **NP10:** Animals treated with 5 nm PVP-AgNP at 10 mg/kg bw. Values are presented as mean ± SEM, n ≥ 5 per group. **p < 0.01 when compared to control (untreated animals)

Although it showed subtler changes, the colonic submucosa of 3/5 and 4/6 of the animals' dose at 1 and 10 mg/kg bw, respectively, presented edema and vascular ectasia phenomena, responsible for an increase in submucosal thickness in the colon (Fig. 4A vs B). This increase was more significant for 10 mg/kg bw-treated animals, which presented a submucosal thickness of 133 ± 7 μm, about 1.5 times larger than the untreated animals (87 ± 10 μm). No significant changes in the thickness of the total intestinal wall, mucosa membrane, and crypt were found.

### Levels of inflammatory cytokines

#### (a) Plasma

Considering the main role of cytokines during an inflammatory response, the levels of some pro-inflammatory (IL-12p70, TNF-α, IFN-γ, MCP-1, IL-6) and anti-inflammatory (IL-10) cytokines were determined in blood plasma. As shown in Table 2, no significant differences were found in the cytokine levels in mice treated with 5 nm PVP-AgNP (either 1 or 10 mg/kg bw) compared to untreated mice, discarding in this way a systemic inflammatory response.

**Table 2:** Levels of inflammatory cytokines and chemokines in the plasma, 14 days after oral administration of 5 nm PVP-AgNP (1 and 10 mg/kg bw)

	Mean Fluorescence Intensity (MFI)		
	Control group	NP1	NP10
IL-12p70	367 ± 7	361 ± 8	362 ± 11
TNF-α	516 ± 20	514 ± 15	514 ± 17
IFN-γ	450 ± 7	437 ± 9	426 ± 11
MCP-1	865 ± 62	799 ± 44	787 ± 32
IL-10	698 ± 21	672 ± 12	677 ± 14
IL-6	963 ± 72	1363 ± 268	853 ± 20 ^#^

Values are presented as mean $\pm$ SEM of n ≥ 6 *per* group. **Control:** Untreated animals; **NP1:** Animals treated with 5 nm PVP-AgNP at 1 mg/kg bw; **NP10:** Animals treated with 5 nm PVP-AgNP at 10 mg/kg bw. ^#^p < 0.05 when compared to mice administered with 1 mg/kg bw of 5 nm PVP-AgNP

#### (b) Colon, ileum, and duodenum homogenates

To understand whether the passage of AgNP through the GIT is reflected by a significative inflammatory activation, the levels of pro-inflammatory (IL-12p70, TNF-α, IFN-γ, MCP-1, IL-6) and anti-inflammatory (IL-10) cytokines were also evaluated in colon, ileum, and duodenum homogenates (Table 3).

**Table 3:** Levels of inflammatory cytokines and chemokines in colon, ileum and duodenum homogenates, 14 days after oral administration of 5 nm PVP-AgNP (1 and 10 mg/kg bw)

	Mean Fluorescence Intensity (MFI)								
	Colon			Ileum			Duodenum		
	Control group	NP1	NP10	Control group	NP1	NP10	Control group	NP1	NP10
IL-12p70	324 ± 20	411 ± 24^*^	371 ± 22	1093 ± 267	1146 ± 248	1087 ± 351	728 ± 164	705 ± 183	682 ± 158
TNF-α	366 ± 16	490 ± 20^**^	464 ± 35^*^	1300 ± 344	1475 ± 334	1345 ± 440	737 ± 149	727 ± 179	712 ± 148
IFN-γ	410 ± 24	474 ± 15	443 ± 31^p=0.09^	1085 ± 251	1123 ± 222	1032 ± 298	775 ± 137	755 ± 165	744 ± 135
MCP-1	615 ± 35	760 ± 13^**^	672 ± 30^#^	1304 ± 252	1338 ± 245	1249 ± 315	919 ± 121	903 ± 165	875 ± 125
IL-10	848 ± 69	646 ± 58^*^	659 ± 35^*^	1694 ± 382	1748 ± 297	1552 ± 439	1100 ± 178	1064 ± 187	1062 ± 161
IL-6	832 ± 14	890 ± 28^p=0.08^	887 ± 14^p=0.09^	1671 ± 294	1667 ± 254	1573 ± 359	1263 ± 167	1261 ± 191	1233 ± 161

Values are presented as mean $\pm$ SEM (MFI) of n ≥ 6 *per* group. **NP1:** Animals treated with 5 nm PVP-AgNP at 1 mg/kg bw; **NP10:** Animals treated with 5 nm PVP-AgNP at 10 mg/kg bw. **p < 0.01, and *p < 0.05 when compared to control (untreated animals)

The results displayed in Table 3 show a significant increase in the levels of inflammatory cytokines in the colon, denoting the occurrence of an inflammatory response caused by exposure to AgNP. In contrast, for the ileum and duodenum, there were no variations in baseline levels of inflammatory cytokines, compared to control groups.

**Fig. 5:**
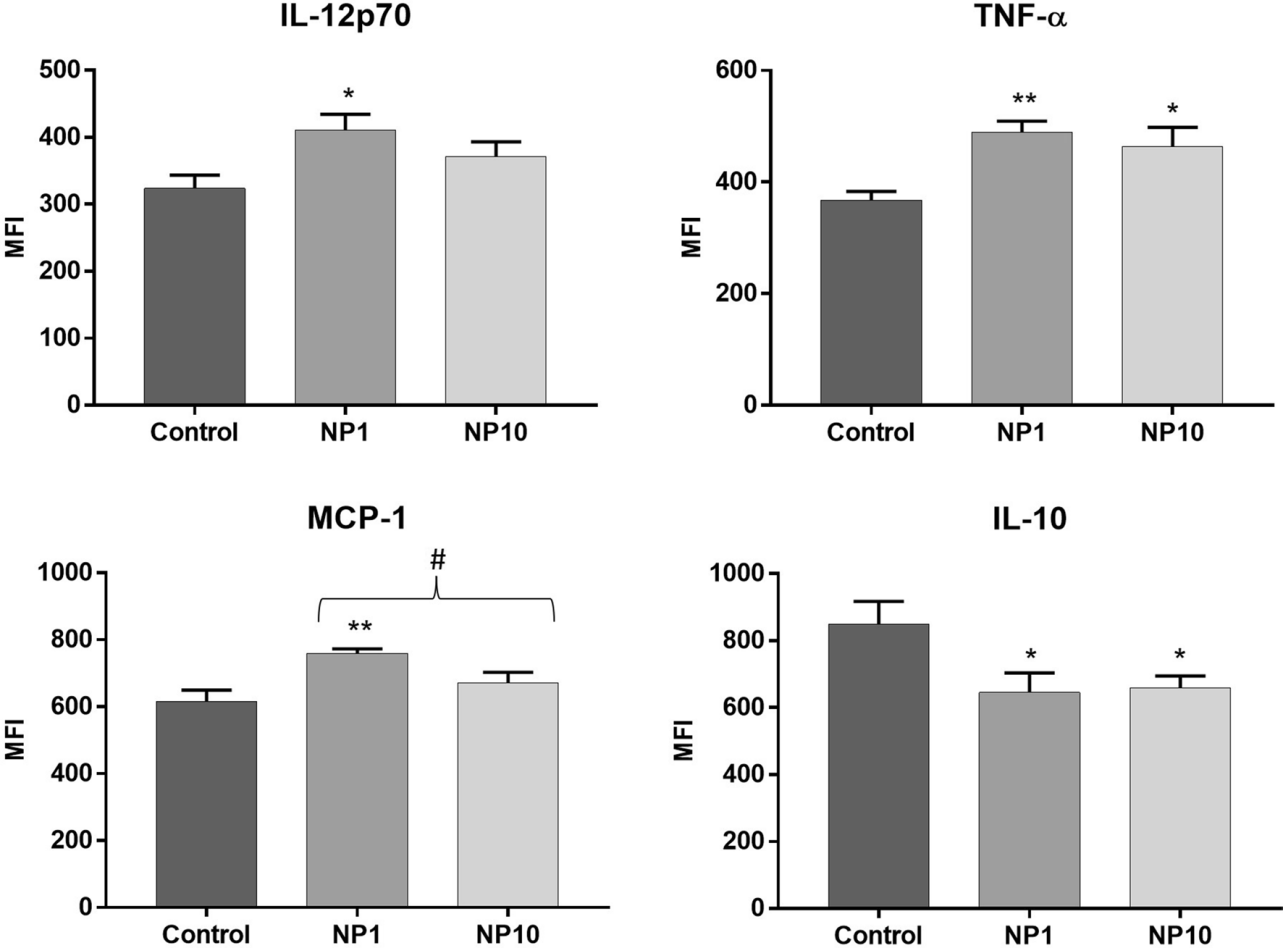
Levels of pro-inflammatory (IL-12p70, TNF-α and MCP-1) and anti-inflammatory (IL-10) cytokines in colon homogenates of C57BL/6J mice, 14 days after oral administration of 5 nm PVP-AgNP (1 and 10 mg/kg bw). **Control:** Untreated animals; **NP1:** Animals treated with 5 nm PVP-AgNP at 1 mg/kg bw; **NP10:** Animals treated with 5 nm PVP-AgNP at 10 mg/kg bw. Values are presented as mean ± SEM (MFI), of n ≥ 6 *per* group. ***p < 0.001 and *p < 0.05 when compared to control (untreated animals)

The colon of mice treated with 5 nm PVP-AgNP exhibited a significant increase in the pro-inflammatory cytokines IL-12p70 and in the chemokine MCP-1, at the lowest administered dose (1 mg/kg bw). For the pro-inflammatory cytokine TNF-α, it was also observed a significant increase for both administered doses (1 and 10 mg/kg bw) (Fig. 5). While the differences in the other pro-inflammatory cytokines, when compared to the controls, did not reach statistical significance, there was a noticeable trend towards an increase in the pro-inflammatory cytokine IFN-γ, for the highest administered dose (10 mg/kg bw), and also in IL-6, for both administered doses (1 and 10 mg/kg bw). As for the levels of the anti-inflammatory cytokine IL-10, there was a significant decrease for both administered doses (Fig. 5).

### Western Blot analysis

Given the presence of inflammatory signals in the cytokine analysis and the observation of mononuclear inflammatory infiltrate, we proceeded to assess the activation of the NF-кB pathway. Its nuclear translocation induces the expression of several pro-inflammatory genes and depends on the phosphorylation and degradation of IкBα. Under basal conditions, IkBα retains NF-kB dimers in the cytoplasm, but after an inflammatory stimulus, IкBα undergoes proteasomal degradation and NF-kB dimers translocate to the nucleus promoting transcription of target genes. Thus, to investigate the effects of 5 nm PVP-AgNP on the transcriptional activation of NF-кB, the expression levels of IкBα and phospho-IкBα in colon, ileum, and duodenum homogenates of treated and untreated mice were evaluated.

**Fig. 6:**
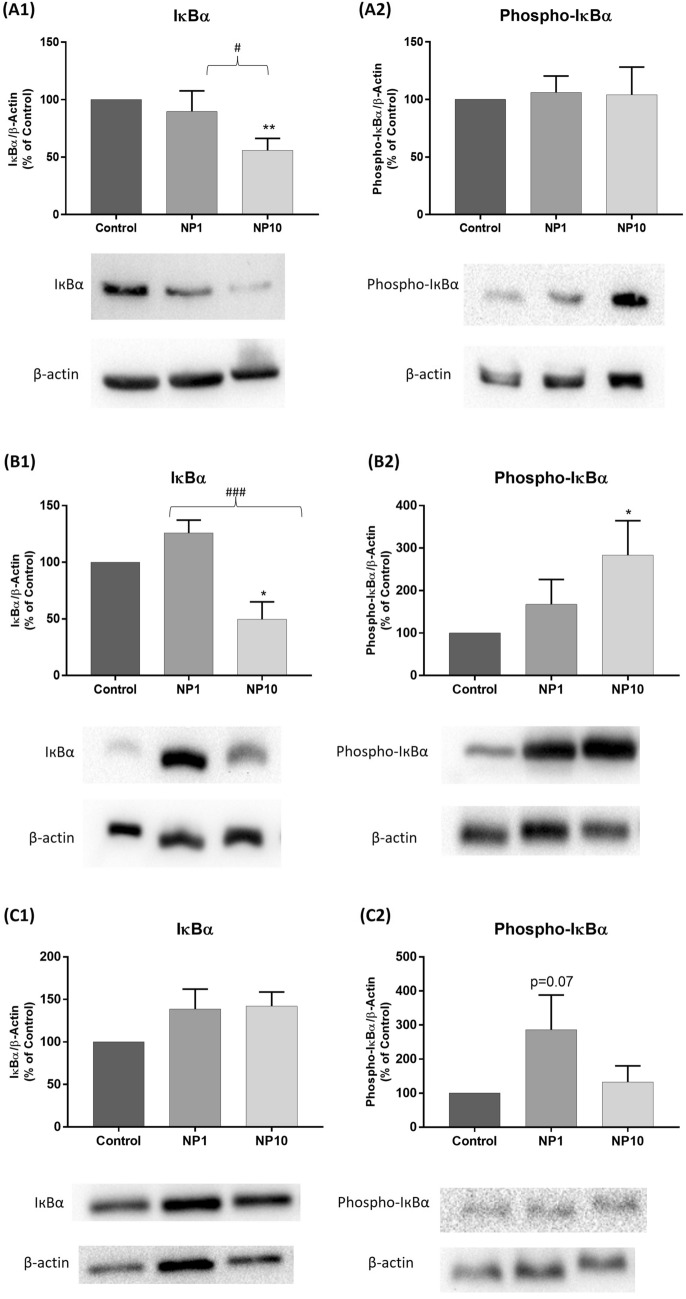
Evaluation of transcriptional activation of NF-кB, through the analysis of the relative proportion of IкBα (MW = 39 kDa) and phospho-IкBα (MW = 40 kDa) in **colon (A1, A2), ileum (B1, B2),** and **duodenum (C1, C2)** homogenates from C57BL/6J mice, 14 days after oral administration of 5 nm PVP-AgNP (1 and 10 mg/kg bw). Below the graphs are represented western blotting bands showing IкBα and phospho-IкBα expression levels. β-actin (MW = 42 kDa) was used for equal loading of protein. **Control:** Untreated animals; **NP1:** Animals treated with 5 nm PVP-AgNP at 1 mg/kg bw; **NP10:** Animals treated with 5 nm PVP-AgNP at 10 mg/kg bw. Values are presented as mean ± SEM of *n* = 6 *per* group. **p < 0.01 and *p<0.05 when compared to control (untreated animals). ###*p*<0.001 and #*p* < 0.05 when compared to mice administered with 1 mg/kg bw of 5 nm PVP-AgNP

Western blotting results for the colon are shown in Fig. 6 (A1, A2) and revealed that the highest dose administered (10 mg/kg bw) induced a significant decrease in the IкBα levels, compared to controls. However, these reduced levels were not accompanied by a significant increase in phospho-IкBα levels.

Similarly, the western blotting analysis of the ileum demonstrated a significant reduction in IкBα levels at the highest administered dose (10 mg/kg bw), which coincided with a notable increase in phospho-IкBα levels at the same dose (Fig. 6B1, B2).

For the duodenum, western blotting revealed no significant changes in IкBα and phospho-IкBα levels in the animals treated with 5 nm PVP-AgNP (either 1 mg/kg bw or 10 mg/kg bw) (Fig. 6 – C1, C2). Nonetheless, the animals treated with the lowest dose of 5 nm PVP-AgNP (1 mg/kg bw) presented a trend (p = 0.07) to an increase in phospho-IкBα levels.

## Discussion

AgNP have become widely present in our daily lives, resulting in exponential and unavoidable oral exposure. As result, several concerns have been raised in recent years about their potential hazards to human health, more precisely their unpredicted harmful and pro-inflammatory effects on the GIT, which must be considered in the risk assessment and risk management of these materials. Despite this increased exposure to AgNP, their adverse effects at the intestinal level are still largely unexplored. Additionally, almost all existing studies on AgNP-induced toxicity were only performed in in vitro models, further limiting the knowledge about their real harmful effects in a more complex organism. Furthermore, the existing in vivo studies mostly use the oral route of administration by gavage, which can be stressful for the animal, interfering with the results obtained. Thus, this study aimed to fill a gap in the literature by evaluating the biological effects of two subacute doses of 5 nm PVP-AgNP in C57BL/6J mice. One mg/kg body weight or 10 mg/kg bw was given once a day for 14 days, using a new technology (HaPILLness) that allows voluntary, stress-free, and accurate oral dosing.

The biodistribution of 5 nm PVP-AgNP was evaluated by measuring Ag levels in various organs/tissues by the ICP-MS technique. The 5 nm PVP-AgNP were shown to translocate throughout the body and accumulate in different organs/tissues in a concentration-dependent manner. Following oral ingestion, AgNP are exposed to a range of conditions and interactions within biological fluids and tissues, often leading to the release of Ag^+^ ions as the most common outcome (Lansdown 2006; Walker and Parsons 2014). In turn, free Ag^+^ ions have a high binding affinity to components of extracellular matrix proteins and other soluble proteins and peptides or even chloride ions in body fluids (Walker and Parsons 2014). Thus, Ag can be absorbed into the systemic circulation, where it is distributed to all organs/tissues of the body (Lansdown 2006; Walker and Parsons 2014). On the other hand, it is possible for a specific quantity of AgNP to evade the typical digestive degradation processes, and subsequently traverse the intestinal mucous layer, ultimately being absorbed in its complete form from the intestinal lumen. Following this, the AgNP may be assimilated into the circulation or lymphatic system through either a paracellular or transcellular pathway (Lu et al. 2021). Although Ag was found in all organs/tissues analyzed, the accumulation was most pronounced in the lungs, followed by the heart, brain, feces, spleen, liver, ileum, colon, heart, and kidneys. Considering that Ag content was also significant in the feces, it can be assumed that a significant percentage of the AgNP ingested is not absorbed by the organism, being consequently excreted by defecation. As ICP-MS enabled the detection Ag but not AgNP, it is crucial to acknowledge that there is no definitive evidence that the observed effects in this study were solely caused by AgNP, as they may also can be attributed to Ag^+^ ions released by AgNP. Recently, Jia et al*.* (2017) reported the biodistribution in C57BL/6J mice orally treated with 30 nm PVP-AgNP solutions at 100 and 300 mg/kg bw for 14 days. The authors also observed the distribution of AgNP in all major organs, namely liver, spleen, lung, heart, kidney, and intestine, with the highest accumulation of Ag in the intestine (Jia et al. 2017). Sofranko et al*.* (Sofranko et al. 2021) studied the effects of 40 nm PVP-AgNP at 400 mg/kg bw on C57BL/6J, which were administered orally to mice in feed pellets ad libitum for 28 days, once a day. The quantification of Ag by ICP-MS revealed that the metal was mainly present in the intestine, followed by the liver and spleen (Sofranko et al. 2021). On the other hand, Bergin et al*.* (Bergin et al. 2016) evaluated the effects of PVP and citrate-coated AgNP (20 nm and 110 nm) by oral gavage in C57BL/6NCrl mice for 3 days, once a day, at a dose of 10 mg/kg bw, and found that most of Ag was present in the feces, with some Ag also detected in the colon, for all AgNP tested. The Ag accumulation in other organs was minimal or similar to the levels in the control group (Bergin et al. 2016). These results, in agreement with those obtained in our study, support the hypothesis that a large part of the AgNP passes through the GI tract and is finally eliminated in the feces. In addition to the animal model used in this study, there have been other studies that have evaluated the biodistribution of AgNP using different mouse models and testing AgNP with different physicochemical properties compared to those used in the present work. While some studies have reported similar findings of higher accumulation in the liver and spleen, others have shown different patterns of accumulation in various organs, such as the lungs, brain, kidneys, heart, and testes. These discrepancies in the results could be attributed to various factors, including the size, coating agent, dose, and duration of exposure to AgNP (Park et al. 2010; Ferdous and Nemmar 2020; Gan et al. 2020, Recordati, De Maglie et al. 2021).

Since the GIT is particularly exposed to and accumulates AgNP, a histological evaluation of eventual changes in the intestinal tissue was performed. The general histological examination demonstrated an intestinal inflammatory response comprising vascular changes (in all intestinal segments studied) and cellular changes (in the duodenum and ileum) in PVP-AgNP-treated animals, regardless of the dose tested, observations that were corroborated by the semiquantitative histomorphometry. Few histological studies have been published that analyze the effect of AgNP in the intestine and none of them carried out a semiquantitative analysis of the histological observations. Shahare et al*.* (Shahare and Yashpal 2013) evaluated the effect of 5–10 nm AgNP (at 5, 10, 15, 20 mg/kg bw *per* day), administered by oral gavage during 21 days, in the small intestine of 8–10-week-old male *Swiss* albino mice. As in our study, the most significant histopathological changes induced by AgNP were a disruption in the border of villi and an increase in the number of inflammatory cells in the lamina propria of each villus, leading to enlargement of the lamina propria (Shahare and Yashpal 2013). Additionally, the authors also observed an increased number of mitotic figure along with dilated intestinal glands, suggesting that AgNP damage to epithelial cells induces stem cell proliferation intending to replace lost enterocytes (Shahare and Yashpal 2013). Therefore, it is possible to infer that, during their passage through the intestine, AgNP damage the intestinal mucosa, triggering a local inflammatory response characterized by an increase in inflammatory cells, accompanied by an increase in mitotic activity, possibly as an attempt to restore the physiological state of the intestine. It is noteworthy that, despite the lack of significant Ag accumulation in the duodenum, it was found to be the intestinal region most affected by the passage of AgNP. This observation reinforce the idea that the degree of AgNP accumulation in the duodenum is influenced by various factors, such as nanoparticle size, coating, dose, and the duration of exposure. In this study, we showed that the duodenum was merely a site of AgNP transit, rather than accumulation/excretion, yet the mere transit of AgNP provoked a notable inflammatory reaction. Jeong et al*.* (Jeong et al. 2010) also studied the histological changes in the intestinal mucosa of four-week-old male and female Sprague–Dawley rats after oral exposure to 60 nm AgNP at 30, 300, and 1000 mg/kg *per* day for 28 days. These authors also found the accumulation of AgNP in the ileum and colon, as in our study (Jeong et al. 2010). In the ileal mucosa, these authors observed a greater number of disordered goblet cells, which released their mucus material into the crypt lumen and into the ileal lumen, but not into the proximal colon (Jeong et al. 2010).

It is currently accepted that cytokines and chemokines are key mediators of cellular interaction in the intestine and play a major role in driving intestinal inflammation (Friedrich et al. 2019; Guan 2019). Studies in the literature evaluating the effect of AgNP on the production of inflammatory cytokines, either in the blood or in the intestine, are very scarce. In our study, cytokines levels of IL-6, IL-10, MCP-1, IFN-γ, TNF-α, and IL-12p70 in plasma and intestinal tissue after exposing mice to 5 nm PVP-AgNP (1 and 10 mg/kg bw) were investigated by flow cytometry. No differences were found in plasma cytokine levels of AgNP-treated mice compared to the untreated controls. In opposition to our results, Park et al*.* (Park et al. 2010), in their study on the effects of prolonged oral exposure (28 days) of ICR mice to 42 nm AgNP (0.25, 0.5 and 1 mg/kg bw *per* day) observed an increase in the cytokines IL-1, IL-6, IL-4, IL-10, IL-12 and tumor growth factor (TGF)-β levels, in a dose-dependent manner, in the serum (Park et al. 2010). These differences may be due to the size of the AgNP, which were larger than the one used in our study, as well as the period of exposure to AgNP that was shorter in our study, which may not be sufficient to trigger a significant production of inflammatory cytokines. Given the significant accumulation of Ag in the intestinal tissue, as well as the histological changes observed in this tissue, the effects of 5 nm PVP-AgNP (1 and 10 mg/kg bw *per* day) on the induction of an intestinal pro-inflammatory response were evaluated, through modulation of the inflammatory cytokine profile. Our results show an inflammatory response in the colon, mainly characterized by a significant increase in the pro-inflammatory cytokines IL-12p70, TNF-α, and chemokine MCP-1, in addition to a significant decrease in the anti-inflammatory cytokine IL-10. Additionally, a trend towards the increase of other pro-inflammatory cytokines like IFN-γ and IL-6 was also observed. Orr et al*.* (Orr et al. 2019) also measured the levels of TNF-α in intestinal tissue, specifically in the ileum of Sprague–Dawley rats exposed to 10 or 100 nm AgNP (9 mg/kg bw) by oral gavage, once a day, for 13 weeks. The authors also reported the occurrence of a pro-inflammatory response through increased TNF-α levels, which was more prominent in animals treated with smaller AgNP (10 nm) than with larger (100 nm) (Orr et al. 2019).

NF-кB is one of the most important transcription factors of the inflammatory pathway, being strongly induced during the orchestration of an intestinal inflammatory response. It is crucial for the regulation and initiation of transcription of several cytokines in the nucleus (Liu et al. 2017; Peng et al. 2020; Chawla et al. 2021). To better elucidate the mechanism of action behind the increase in the levels of inflammatory cytokines observed in our study, the NF-кB expression levels were analyzed by Western Blot in colon, ileum, and duodenum homogenates. In the colon, IкBα expression significantly decreased for the highest dose tested (10 mg/kg bw). Despite this, the phospho-IкBα expression remained unchanged. Interestingly, in the ileum, where changes in cytokines and chemokines levels were not observed, a significant decrease in IкBα expression was observed, which was accompanied by an increase in phospho-IкBα expression. In the duodenum, the levels of both proteins remained unchanged with the administration of AgNP. Taken globally, these results allow us to conjecture that the passage of AgNP through the colon triggers the development of an inflammatory response that involves the activation of the NF-кB pathway and culminates in the production of cytokines and chemokines. On the other hand, in the ileum, the activation of the NF-кB inflammatory pathway seems not to be the main responsible for  the production of cytokines and chemokines, probably being involved in the production of other inflammatory signals (Sousa et al. 2022). As far as we know, there are no studies in the literature that relate the NF-кB activation with AgNP-induced cytotoxicity at the intestinal level. However, its activation is known to be implicated in the pathogenesis of IBD. Evidence indicates that the NF-κB pathway is upregulated in the gut mucosa of individuals with IBD, and that dysregulation of this signaling pathway can lead to chronic inflammation, tissue damage, and impaired healing. Therefore, the NF-κB pathway is considered to be a critical mediator of IBD pathology (NEURATH et al. 1998; Mitsuyama et al. 2001; Peng et al. 2020; Chawla et al. 2021; Sousa et al. 2022). Multiple studies have indicated that exposure to AgNP can result in the activation of the NF-κB pathway across various cell types (Eom and Choi 2010; Nishanth et al. 2011; AshaRani et al. 2012; Kapka-Skrzypczak et al. 2020). This activation leads to an increase in the expression of pro-inflammatory cytokines and other downstream effects. Given these findings, it appears that the NF-κB pathway is an important target for investigating the mechanisms of toxicity and the potential health effects of AgNP.

## Conclusions

This study constitutes an in vivo approach to the biodistribution and potential pro-inflammatory effects of 5 nm PVP-coated AgNP in mice. As a peculiarity of the study, the use of a new oral dosing technology (HaPILLness) that allows voluntary, stress-free, and accurate dosage should be highlighted.

Once inside the body, AgNP can interact with biological fluids and tissues, leading to various transformations, such as dissolution and agglomeration. Although a portion of AgNP can remain intact and maintain their size and shape while translocating in the organism, they can also release Ag^+^ ions. The results robustly showed that, after oral ingestion, AgNP/Ag^+^ reach the bloodstream and are distributed throughout the body, including the intestine, and excreted in the feces. The contact of AgNP/Ag^+^ ions with the intestine triggers a local inflammatory response, translated into significant vascular and cellular changes, as well as the activation of the fundamental inflammatory NF-кB pathway, culminating in the production of several cytokines and chemokines.

Considering the paucity of literature on this subject, our results provide new and important information on subacute in vivo toxicity and distribution of AgNP. However, more research is needed for a robust characterization of AgNP toxicity in living organisms with a view to their safe application in the food industry and other fields.

## Data Availability

Data are available from the corresponding author upon request.
